# Prevalence and determinants of essential newborn care practices in the Lawra District of Ghana

**DOI:** 10.1186/s12887-018-1145-4

**Published:** 2018-05-24

**Authors:** Mahama Saaka, Fusena Ali, Felicia Vuu

**Affiliations:** University for Development Studies, School of Allied Health Sciences, P O Box, 1883 Tamale, Ghana

**Keywords:** Essential newborn care, Newborn danger signs, Neonate feeding, Optimal thermal care, Safe cord care, Lawra District

## Abstract

**Background:**

There was less than satisfactory progress, especially in sub-Saharan Africa, towards child and maternal mortality targets of Millennium Development Goals (MDGs) 4 and 5. The main aim of this study was to describe the prevalence and determinants of essential new newborn care practices in the Lawra District of Ghana.

**Methods:**

A cross-sectional study was carried out in June 2014 on a sample of 422 lactating mothers and their children aged between 1 and 12 months. A systematic random sampling technique was used to select the study participants who attended post-natal clinic in the Lawra district hospital.

**Results:**

Of the 418 newborns, only 36.8% (154) was judged to have had safe cord care, 34.9% (146) optimal thermal care, and 73.7% (308) were considered to have had adequate neonatal feeding. The overall prevalence of adequate new born care comprising good cord care, optimal thermal care and good neonatal feeding practices was only 15.8%.

Mothers who attained at least Senior High Secondary School were 20.5 times more likely to provide optimal thermal care [AOR 22.54; 95% CI (2.60–162.12)], compared to women had no formal education at all. Women who received adequate ANC services were 4.0 times (AOR  =  4.04 [CI: 1.53, 10.66]) and 1.9 times (AOR  =  1.90 [CI: 1.01, 3.61]) more likely to provide safe cord care and good neonatal feeding as compared to their counterparts who did not get adequate ANC. However, adequate ANC services was unrelated to optimal thermal care.

Compared to women who delivered at home, women who delivered their index baby in a health facility were 5.6 times more likely of having safe cord care for their babies (AOR = 5.60, Cl: 1.19–23.30), *p* = 0.03.

**Conclusions:**

The coverage of essential newborn care practices was generally low. Essential newborn care practices were positively associated with high maternal educational attainment, adequate utilization of antenatal care services and high maternal knowledge of newborn danger signs. Therefore, greater improvement in essential newborn care practices could be attained through proven low-cost interventions such as effective ANC services, health and nutrition education that should span from community to health facility levels.

**Electronic supplementary material:**

The online version of this article (10.1186/s12887-018-1145-4) contains supplementary material, which is available to authorized users.

## Background

Many countries especially in sub-Saharan Africa were unable to achieve the Millennium Development Goals (MDGs) 4 and 5. In particular, the set target of reducing under-five mortality rates by two-thirds as of 2015 was not met because there was little reduction in neonatal mortality (NMR). Many countries are reported to have made little or no progress towards the child survival target, and that some countries in sub-Saharan Africa had even witnessed a deterioration in child survival rates [1].

Suboptimal newborn care practices still persist and neonatal mortality rates have been resistant to change and now contribute about 40% of all under-five deaths world-wide [2]. In Ghana, neonatal mortality is estimated at 29 /1000 live births and 68% of all deaths among children under age five years take place before a child’s first birthday, with 48% occurring during the first month of life [3]. Though there has been a decline of 29% in neonatal mortality since 1993 in Ghana, the decrease was at a slower pace than infant and child mortality. Consequently, the contribution of neonatal deaths to infant deaths had increased from 53% in 1998 to 71% in 2014 [3].

World leaders have launched a renew effort to reduce child mortality under the Sustainable Development Goals (SDGs). One major target of the SDGs is to “end preventable deaths of newborns and children under 5 years of age, with all countries aiming to reduce neonatal mortality to at least 12 per 1,000 live births and under-5 mortality to at least 25 per 1,000 live births by 2030” [4]. To achieve this target, it is important for mothers to adopt essential newborn care practices. To help reduce newborn morbidity, mortality and promote healthy newborn care practices, the World Health Organization (WHO) recommended a package of essential newborn care practices some of which include clean cord care (that is, cutting and tying of the umbilical cord with a sterilized instrument and thread), thermal care (drying and wrapping the newborn immediately after delivery and delaying the newborn’s first bath for at least 24 h or several days to reduce hypothermia risk), and initiating breastfeeding within the first hour of birth [5].

It has been estimated globally that over two-thirds of newborns could be saved through existing maternal and child health programmes that relate to cord care to decrease sepsis, temperature control and initiation of early breastfeeding [6–10].

In Ghana, skilled assistance during delivery has increased from 40% in 1988 to 74% in 2014. Facility-based deliveries have increased from 42% in 1993 to 73% in 2014. Despite this modest improvement, more than one-quarter of births occur at home especially in rural areas. In the Upper West Region where this study was conducted, delivery in a health facility was 63% of births [3].

Deliveries at home are usually without the support of skilled birth attendants (SBA). Under such circumstances, babies may not receive the recommended immediate newborn care practices and so become more vulnerable to infection-related risks [11]. There is evidence that suggests that most neonatal deaths in developing countries occur at home, and attended by unskilled health practitioners [2]. However, very little is documented on the traditional newborn care practices in the Lawra District of Ghana where a significant number of deliveries still take place at home. It is in view of this that this study was undertaken. The main aim was to describe the prevalence and determinants of essential newborn care practices.

## Methods

### Study setting

The study was carried out in the Lawra District hospital. The Lawra District lies in the North-West corner of the upper West Region of Ghana. The total area of the District is 1051.2 km^2^. The District has two hospitals located in Lawra and Nandom. They provide clinical and public health services as well as serve as a referral centres for the sub-districts. There are 10 sub-districts which provide primary health care services.

Apart from agriculture, which engages about 80% of the population, there are small scale enterprises such as petty trading, artisanal works, small-scale industry enterprises, hotel/restaurants/chop bar and transport services. There are also those employed as public servants, although wages are low. The dominant economic activity is agriculture which does not yield the required returns necessary for meaningful standards of living. The result is wide spread poverty among the people with severe impact on women and children.

### The study design, population, sampling

A cross-sectional study was carried out in June 2014 on a sample of 422 lactating mothers and their children. The primary study population comprised women of reproductive age (15 to 49 years) who have delivered a live baby within the past 12 months prior to the conduct of this study. The 12-month limit was set with the intention of mitigating recall bias by the mother. A systematic random sampling technique was used to select the study participants who attended post-natal clinic in the Lawra District Hospital. The list of mothers contained in the attendance register for mothers who sought post-natal care served as the sampling frame. A sampling interval was calculated by dividing the total number of mothers (800) by the required sample size of 422. A random number between 1 and the sampling interval was selected to be the starting point of the sample extraction. Subsequently, the study participants were selected by adding the sampling interval to the number corresponding to the previous mother chosen on the list. This process was continued until the required number was obtained.

A sample size of 384 was required to ensure that the estimated prevalence of the main outcome variable (coverage of essential new born care practices) was within plus or minus 5% of the true prevalence at 95% confidence level. An additional 10% to adjust for unexpected events (e.g. damaged/incomplete questionnaire) was factored in the sample size determination and so the sample size was 422.

### Data collection

A structured questionnaire was administered through face to face interview to obtain information from respondents. The questionnaire comprised different sections including socioeconomic and demographic information, birth preparedness, knowledge of women about newborn danger signs, care during pregnancy and delivery (Additional file 1).

#### Dependent and independent variables

The women were asked questions on essential newborn care practices including a) type of instrument used to cut the umbilical cord b) whether the newborn was dried and wrapped soon after delivery, c) the number hours or days after birth the newborn was first bathed d) the temperature of the water used in bathing e) whether any pre-lacteal food or drink was given, and f) the number of hours or days after birth breastfeeding was initiated g) whether colostrum was fed to the baby h) whether exclusive breast feeding was practiced.

Three composite indices of essential newborn care practices (safe cord care, optimal thermal care and good neonatal feeding practices) were the main outcome measures used in the study.

Safe cord care was defined as use of a clean cutting instrument to cut the umbilical cord plus clean thread to tie the cord plus no substance applied to the cord. Optimal thermal care was defined as baby wrapped within 10 min of birth plus first bath after 6 or more hours plus using warm water to bath the baby. A child was considered to have received good neonatal feeding, he/she should be breast feeding at the time of the study, initiated breastfeeding within the first 1 hour after birth, not being fed with prelacteals, fed with colostrum and avoidance of bottle-feeding. If one or more of the conditions were not met, then the feeding practice was described as inadequate or bad.

The independent variables included socio-demographic factors, maternal age*,* educational attainment, ethnicity, religion etc. Socio-economic statu*s* (SES) was measured as household wealth index. Principal components analysis (PCA) was used to quantify a proxy measure of SES based on ownership of specified durable goods (television, radio, car, mobile telephone, etc.) and housing characteristics (access to electricity, source of drinking water, type of toilet facilities, type of flooring material and type of cooking fuel) [12]. Utilization of antenatal care services and maternal knowledge on newborn danger signs were also assessed as explanatory variables.

### Data analysis

Descriptive and inferential statistics were done using the predictive analytic software (PASW) for Windows version 18.0 and statistical significance was taken when *p* < 0.05. Chi-square statistics were performed to compare the levels of each of the dependent variables with the explanatory variables. A multiple logistic regression was used to identify socio-demographic, mothers’ knowledge of specific newborn danger signs, attendance at delivery by skilled birth attendant, antenatal and delivery care factors that were associated with the three newborn care practices (that is, safe cord care, optimal thermal care and good neonatal breastfeeding). Explanatory variables which were significant at bivariate analysis at a *p*-value of 0.05 or less were fed into the regression model after confirming the absence of multi-collinearity between these independent variables.

### Ethical considerations

The study protocol was approved by the Scientific Review and Ethics Committee of the School of Allied Health Sciences, University for Development Studies, Ghana.

Informed consent was also obtained after needed information and explanation. In situations, where the respondent could not write or read, verbal informed consent was sought from all the study participants before the commencement of any interview. Data were analyzed and presented anonymously.

## Results

In all 422 women were recruited for the study, but due to incomplete responses to questionnaires, the valid responses obtained from respondents was 418, giving a response rate of 99.1%.

### Socio-demographic characteristics of study sample

The 418 respondents were mothers who were residents in rural settings. The mean age was 28.5 (SD 5.6) years with a range of 16–49 years. The details of the sample characteristics including maternal educational level, marital status, religion of mothers, occupation of mothers, and maternal age distribution are shown in Table 1. About 29 (6.9%) of the mothers had no formal education and 75 (17.9%) attained tertiary level of education. The majority of the mothers 333 (79.7%) were married. Most of the mothers 239 (57.2%) were Christians. Most 182 (43.5%) of the mothers were petty traders and only 18 (4.3%) mothers were farmers. More than half 263 (62.9%) of the infants were females.

**Table 1:** Socio-demographic and reproductive characteristics of respondents (*N* = 418)

Age category	Frequency *n* (%)
Under 20 years	14 (3.3)
20–34 years	349 (83.5)
At least 35 years	55 (13.2)
Religion	
Christianity	239 (57.2)
Islam	179 (42.8)
Marital Status	
Married	333 (79.7)
Single	81 (19.4)
Divorced	1 (0.2)
Widowed	3 (0.7)
Educational Level	
None	29 (6.9)
Primary	54 (12.9)
JHS	113 (27.0)
SHS/Vocational/Technical	147 (35.2)
Tertiary	75 (17.90
Occupational Level	
Unemployed	69 (16.5)
Petty trader	182 (43.5)
Farmer	18 (4.3)
Civil / Public Servant	50 (12.0)
Others	99 (23.7)
Maternal Knowledge of dangers of newborn care signs	
1–3	229 (54.8)
At least 4	189 (45.2)
Total	418 (100.0)
Gravidity	
Primigravidae	166 (39.7)
Secundigravidae	132 (31.6)
Multigravidae	120 (28.7)
Parity	
Primiparous	211 (50.5)
Secundiparous	111 (26.6)
Multiparous	96 (23.0)
Timing of first ANC visit	
First trimester	372 (89.0)
Second trimester	41 (9.8)
Third trimester	5 (1.2)

### Coverage of essential newborn care practices

Of the 418 respondents, 215 (51.4%) reported their babies were wrapped 5 min after delivery and 12(2.9%) were wrapped between 30 and 60 min after birth. In 85.4% of the deliveries, scissors were used to cut the cord and 39.7% (166) of the babies were bathed within 1–6 h of delivery. Shea- butter was commonly applied to the umbilical stump in 60.3% (252) of the babies and 51.4% were wrapped immediately after delivery. The main reasons given by mothers for applying substances to the cord stump were to prevent infection and aid healing (Table 2).

**Table 2:** Newborn Care Practices (*N* = 418)

New born care practice	Frequency *n* (%)
Instrument used to cut the umbilical cord	
New blade	48 (11.5)
Any available blade	5 (1.2)
Scissors	357 (85.4)
Others	8 (1.9)
Total	418 (100)
Material used to clamp tie cord	
Thread	18 (4.3)
Cord tie	14 (3.3)
Cord clamp	385 (92.1)
Others	1 (0.2)
Total	418 (100.0)
What was applied to cord	
Nothing	12 (2.9)
Shea butter	252 (60.3)
Spirit	147 (35.2)
Shea butter with powder	3 (0.7)
String	1 (0.2)
Others	3 (0.7)
Total	418 (100.0)
Time baby was wrapped	
Immediately (< 5 min)	215 (51.4)
5–10 min	155 (37.1)
30–60 min	12 (2.9)
Unknown	36 (8.6)
Total	418 (100.0)
Timing of newborn’s first bath	
Soon after birth	29 (6.9)
1–6 h	166 (39.7)
More than 6 h but less than 24 h	148 (35.4)
More than 24 h	4 (1.0)
Can’t tell	71 (17.0)
Total	418 (100.0)
Reasons for applying substances to the cord stump	
Prevent infection	109 (26.1)
To aid healing	281 (67.2)
Keep it dry	4 (1.0)
Prevent water from entering the stomach	8 (1.9)
Midwifes advised me to used	8 (1.9)
Not Applicable	8 (1.9)
Total	418 (100.0)

Three composite indices of essential newborn care practices (safe cord care, optimal thermal care and good neonatal feeding practices) were created. The coverage of these composite newborn care measures was generally low. Of the 418 newborns, only 36.8% (154) were judged to have had safe cord care, 34.9% (146) optimal thermal care, and 73.7% (308) were considered to have had adequate neonatal feeding. The overall prevalence of adequate newborn care comprising good cord care, optimal thermal care and good neonatal feeding practices was only 15.8% (Table 3).

**Table 3:** Composite measures of newborn care practices

New born care practice	Frequency *n* (%)
Safe cord care	
No	264 (63.2)
Yes	154 (36.8)
Total	418 (100.0)
Optimal thermal care	
No	272 (65.1)
Yes	146 (34.9)
Total	418 (100.0)
Good neonatal feeding	
No	110 (26.3)
Yes	308 (73.7)
Total	418 (100.0)
Adequate total neonatal care	
No	352 (84.2)
Yes	66 (15.8)
Total	418 (100.0)

Women who were classified as high ANC content had a significantly higher prevalence of good infant feeding compared to women who received low ANC content (75.5% versus 58.7%) (Chi-squared = 6.0, *p* = 0.014). Similarly, women whose ANC attendance was adequate were more likely to provide optimal thermal care (37.9% versus 10.9%) (Chi-squared = 13.2, *p* < 0.001). Adequate ANC attendance was also associated with safe cord care (χ2 = 15.0, *p* < 0.001).

### Determinants of essential newborn care practices

We tested whether the composite newborn care practices (safe cord care, optimal thermal care and good neonatal feeding) were related to socio-demographic factors, socio-economic status, and adequacy of antenatal services and mothers’ knowledge of specific newborn danger signs.. The predictors were common for the newborn care practices. Overall, the findings show that good neonatal feeding practices, optimal thermal care and good cord care were commonly adopted among women aged 25–34 years, women who had adequate ANC attendance (that is, ANC early initiated in first trimester and at least 4 ANC visits), and those who could mention at least 4 danger signs of the newborn (Tables 4, 5, 6).

**Table 4:** Predictors of good cord care (Bivariate analysis)

		Good cord care?		
Variable	*N*	No *n* (%)	Yes *n* (%)	Test statistic
Age (years)				
Under 20 years	14	5 (35.7)	9 (64.3)	Chi-square (*χ*^*2*^) = 11.6, *p* = 0.003
20–34 years	349	215 (61.6)	134 (38.4)	
At least 35 years	55	44 (80.0)	11 (20.0)	
Adequacy of ANC attendance				
No	46	41 (89.1)	5 (10.9)	Chi-square (*χ*^*2*^) = 15.0, p < 0.001
Yes	372	223 (59.9)	149 (40.1)	
Knowledge of dangers of newborn care signs				
1–3	229	156 (68.1)	73 (31.9)	Chi-square (χ^2^) = 5.4, *p* = 0.02
At least 4	189	108 (57.1)	81 (42.9)	
Mothers’ education				
None	29	25 (86.2)	4 (13.8)	Chi-square (χ^2^) = 14.0, *p* = 0.001
Low	167	115 (68.9)	52 (31.1)	
High	222	124 (55.9)	98 (44.1)

**Table 5:** Relationship between optimal thermal care and socio-demographic/antenatal care factors (*N* = 418)

		Optimal thermal care?		
Variable	*N*	No *n* (%)	Yes *n* (%)	Test statistic
Age (years)				
Under 20 years	14	10 (64.3	5 (35.7)	Chi-square (*χ*^*2*^) = 10.1, *p* = 0.006
20–34 years	349	216 (61.9)	133 (38.1)	
At least 35 years	55	46 (83.6)	9 (16.4)	
Total				
Mothers education				
None	29	24 (82.8)	5 (17.2)	Chi-square (χ^2^) = 45.4, *p* < 0.001
Low	167	131 (78.4)	36 (21.6)	
High	222	113 (50.9)	109 (49.1)	
Religion of mother				
Christianity	239	167 (69.9)	72 (30.1)	*χ*^*2*^ = 5.7, *p* = 0.02
Islam	179	105 (58.7)	74 (41.3)	
Knowledge of newborn danger signs				
1–3	229	165 (72.1)	64 (27.9)	*χ*^*2*^ = 10.9 *p* = 0.001
At least 4	189	107 (56.6)	82 (43.4)	
Adequacy of ANC attendance				
No	46	41 (89.1)	5 (10.9)	Chi-square (χ^2^) = 13.2, *p* < 0.001
Yes	372	231 (62.1)	141 (37.9)

**Table 6:** Relationship between good neonatal feeding and socio-demographic/antenatal care factors (*N* = 418)

		Good neonatal feeding?		
Variable	N	No n (%)	Yes n (%)	Test statistic
Age (years)				
Under 20 years	14	5 (35.7)	9 (64.3)	Chi-square (*χ*^*2*^) = 6.2, *p* = 0.05
20–34 years	349	85 (24.4)	264 (75.6)	
At least 35 years	55	22 (40.0)	33 (60.0)	
Adequacy of ANC attendance				
No	46	19 (41.3)	27 (58.7)	Chi-square (χ^2^) = 5.9, *p* = 0.014
Yes	376	91 (24.5)	281 (75.5)	
Mothers’ education				
None	29	10 (34.5)	19 (65.5)	*χ*^*2*^ = 5.5, *p* = 0.06
Low	167	52 (31.1)	115 (68.9)	
High	222	48 (21.6)	174 (78.4)	
Knowledge of newborn danger signs				
1–3	229	74 (32.3)	155 (67.7)	*χ*^*2*^ = 9.4 *p* = 0.002
At least 4	189	36 (19.0)	153 (81.0)	

In logistic regression analysis, the main predictors of essential new born care practices were maternal educational attainment, maternal age, and adequacy of antenatal care (ANC) attendance and maternal knowledge of newborn danger signs.

Maternal age at interview showed an independent association with essential newborn care practices. Compared to women aged at least 35 years, women under 20 years were 6.6 times more likely to provide safe cord care to their babies (AOR = 6.57, Cl: 1.71–25.31), *p* = 0.006. Compared to women aged at least 35 years, their counterparts aged 20–34 years were 3.3 times (AOR  =  3. 28 [CI: 1.34, 8.04]), 2.0 times (AOR  =  2.02 [CI: 1.11, 3.70]) and 7.3 times (AOR  = 7.31 [CI: 1.65, 32.44]) more likely to provide optimal thermal care, good neonatal feeding and overall adequate new born care practices respectively (Table 7). Compared to women who delivered at home, women who delivered their index baby in a health facility were 5.6 times more likely to have safe cord care for their babies (AOR = 5.60, Cl: 1.19–23.30).

**Table 7:** Determinants of safe cord care, optimal thermal care and good neonatal feeding

Variable	AOR (95% CI) safe cord care	AOR (95% CI) optimal thermal care	AOR (95% CI) good neonatal feeding	AOR (95% CI) ^a^Overall adequacy of new born care practices
Maternal Age (years)				
≥ 35	Reference	Reference	Reference	Reference
Under 20 years	6.57 (1.71, 25.31)^**^	3.65 (0.79, 16.86)	2.22 (0.55, 9.0)	7.41 (0.85, 64.91)
20–34 years	2.01 (0.98, 4.12)	3.28 (1.34, 8.04) ^**^	2.02 (1.11, 3.70)^*^	7.31 (1.65, 32.44)^**^
Adequacy of ANC attendance				
No	Reference	Reference	Reference	Reference
Yes	4.04 (1.53, 10.66)^**^	2.74 (1.01, 7.55)	1.90 (1.01, 3.61)^*^	Not significant
Place of delivery				
Home	Reference	Reference	Reference	Reference
Health facility	5.60 (1.19, 26.30)^**^	Not significant	Not significant in final model	Not significant in final model
Mother could recall at least 4 new born danger signs?				
1–3	Reference	Reference	Reference	Reference
At least 4	Not significant in final model	Not significant	2.00 (1.26, 3.17) ^**^	2.51 (1.41, 4.47)^**^
Maternal Educational level				
No education	Reference	Reference	Reference	Reference
Low (Primary to JHS)	Not significant in final model	6.76 (0.85, 53.89)	Not significant in final model	Not significant in final model
High (At least SHS)	Not significant in final model	20.54 (2.60, 162.12)^**^	Not significant in final model	Not significant in final model
Type of religion				
Christianity	Reference	Reference	Reference	Reference
Islam	Not significant in final model	1.72 (1.09, 2.72)^*^	Not significant in final model	Not significant
Parity				
Primiparous	Reference	Reference	Reference	Reference
Secundiparous	Not significant	1.19 (0.70, 2.02)	Not significant	2.05 (1.08, 3.89)^*^
Multiparous	Not significant	2.67 (1.41, 5.05)^**^	Not significant	2.04 (0.97, 4.30)

AOR (95% CI): Adjusted odds ratio at 95% confidence level

^*^significant at *p* < 0.05; ^**^significant at *p* < 0.01; ^***^significant at *p* < 0.001

^a^Overall adequacy of new born care practices was defined to comprise safe cord care, optimal thermal care and good neonatal feeding

Women who received adequate ANC services were 4.0 times (AOR  =  4.04 [CI: 1.53, 10.66]) and 1.9 times (AOR  =  1.90 [CI: 1.01, 3.61]) more likely to provide safe cord care and good neonatal feeding as compared to their counterparts who did not get adequate ANC. However, adequate ANC services was unrelated to optimal thermal care.

Compared to primiparous women, multiparous women were 2.7 times (AOR  =  2.67 [CI: 1.41, 5.05]) more likely to provide optimal thermal care whilst secondi-parous were 2 times (AOR  =  2.05 [CI: 1.08, 3.89]) more likely to provide overall adequate new born care.

Mothers who attained at least Senior High Secondary School were 20.5 times more likely to provide optimal thermal care [AOR 22.54; 95% CI (2.60–162.12)], compared to women had no formal education at all.

Maternal knowledge of at least four key newborn danger signs was significantly associated with increased probability of providing good neonatal feeding to babies (AOR = 2.0, 95% CI: 1.26–3.17). Furthermore, mothers who could recall at least 4 new born danger signs were at least 2 times more likely to provide overall adequate new born care practices than their counterparts who knew less than 4 new born danger signs.

## Discussion

This study is the first that has assessed the prevalence and determinants of newborn care practices in the Lawra District of Ghana. Though some studies conducted in Ghana have highlighted on optimal thermal care and clean delivery practices for newborns [13–15], issues regarding neonatal feeding practices have received less attention. Furthermore, this present study has used composite indices which give a better reflection of safe cord, optimal thermal care and neonatal feeding. The findings of our study remain relevant because they confirm that of earlier studies and also fill a knowledge gap by showing that using single practice indicators could be less informative in the magnitude of essential newborn practices. For example, optimal thermal care as assessed in the present study included summing up scores for wrapping the baby within 10 min of birth, delaying for at least 6 h and using warm water. A study that uses only one of these indicators will present a different situation from one that uses a combination of these variables.

### Prevalence of essential newborn care practices

Three composite newborn care practices (safe cord care, optimal thermal care, and good neonatal breastfeeding) were investigated and the coverage of these was found to be generally low. These composite variables give a better reflection of safe cord, optimal thermal care and neonatal feeding. The three newborn care practices were associated with socio-demographic, antenatal and delivery care factors.

As in other studies, safe cord care was defined as use of a clean cutting instrument to cut the umbilical cord plus clean thread to tie the cord plus no substance applied to the cord [16–18]. Cord cutting with safe instruments such as new razor blades and scissors was commonly practiced (96.9%) but then when one takes a closer and more holistic approach to safe cord care (that is using composite indicators), only 36.8% actually received that. This means assessing the situation using single practice indicators could be misleading. Poor cord care was driven mainly by putting substances on the cord.

Most mothers applied various things like shea-butter, methylated spirit, shea-butter mixed with powder to the cord stump. The reasons given for applying substances to the cord stump include hastening the healing process, prevent cord stump from smelling and infections and also to prevent water from entering into newborn’s stomach. Since these substances are coming from unsterile sources the likelihood of contamination is high and may thus be harmful to newborns. Similar findings have been reported in the Asante Akim North District in Southern Ghana [19] and in Bangladesh where unhygienic cord care practices were prevalent [20].

Optimal thermal care (defined as baby wrapped within 10 min of birth plus first bath after six or more hours plus using warm water to bath the baby) is an essential intervention for the survival of the newborn especially during the first 24 h of birth. Though most babies were wrapped soon after delivery and were also bathed with hot water, poor thermal care was caused mainly by early bathing. In this study, 46.7% of the newborns were given a bath in less than 6 hours of delivery. Apart from exposing newborns to hypothermia, early bathing removes maternal bacteria and the vernix caseosa (a potent inhibitor of *Escherichia coli*) [21], and eliminates the crawling reflex [22]. Aside its role as a protective barrier from liquids while in the uterus, vernix serves as an antioxidant, skin cleanser, moisturizer, temperature regulator, and a natural, safe antimicrobial for the new baby post-delivery [5, 23]. These are some of the reasons why WHO recommended delay bathing up to at least 24 h. This notwithstanding, many societies have some reasons why babies are bathed early after delivery.

For a normal uncomplicated vaginal birth in a health facility, it is recommended that healthy mothers and newborns should receive care in the facility for at least 24 h after birth [5]. In Ghana, women who deliver in health facilities are detained for a minimum of 6 h and not 24 h. Women who for some reasons deliver at home do not even wait for the 6 h before bathing the newborn. This is why in our study, we limited waiting period to at least 6 h. Early bathing of the baby is a cultural practice and so asking for a waiting time of 24 h appears to be something that is impossible at least for now. Our data showed that only 4 (1%) of the newborns in our sample will have received optimal thermal care if the 24-h cutoff was used instead of 6 h.

In an intervention trial in Ghana that used home visits to promote positive behavior change regarding thermal care, only 41% of mothers delayed bathing for more than 6 h in the intervention areas compared to 29% in the comparison areas [24].

The timing of bathing the newborn appears inconsistent as reported in some earlier studies conducted in Ghana [13, 15]. Whereas some mothers reported bathing their newborns shortly after delivery, others mentioned waiting until later in the day but the exact waiting period has not been more than a few hours after delivery. As reported in one of these studies, early bathing was commonly practiced in Ghana as a measure to reduce body odour in later life, shaping the baby’s head, and to make the baby sleep and feel clean [15].

Some studies conducted on thermal practices in many countries including Ghana, Tanzania, Uganda and India have reported strong cultural beliefs in the benefits of early bathing [15, 25–30], which largely promotes the practice. The practice of early bathing is rooted in the firm belief that the birth process is dirty and that the baby is dirty after birth. This is particularly the case if there is an obvious sign of vernix which is regarded as dirty.

The problem of early bathing of the newborn appears to be widespread across the world. A study in Nepal reported that newborn babies are considered dirty since they came out of their mother’s womb, so almost all newborn babies are bathed within the first hour of birth [31]. Another study conducted in low socioeconomic settlements of Karachi, Pakistan, revealed that newborns were bathed immediately after delivery as the vernix was considered “dirty looking” and it was felt it should be removed [32].

A composite index comprising good neonatal feeding showed that more than 70% of respondents practiced adequate feeding behaviours. For example, timely initiation of breastfeeding (TIBF) rate was 97%.

There are a number of benefits associated with early initiation of breastfeeding including stimulation of breast milk production and the release of oxytocin, which helps the contraction of the uterus and reduces post-partum blood loss [33].

The analysis from this study showed that the expected essential newborn care practices are not available to a greater proportion of the newborns. Poor newborn care practices together with poor maternal care and staff shortages in rural health facilities are major contributing factors of newborn mortality in developing countries [34]. The fact that a greater number of newborns are not getting adequate care suggests the need to extend these essential newborn care practices to the rural areas through rigorous training of community health workers in newborn care practices and sustained educational campaigns by the Ghana Health Service and other health related non-governmental agencies.

It is important to note that about 50% of newborn deaths occur within 24 h of birth [2].

Furthermore, though hypothermia contributes to neonatal morbidity and mortality in low-income countries [35, 36], yet the Ghana National Newborn Strategy and Action Plan (2014–2018) is silent on the specifics on the WHO recommendation on delay bathing of the newborn. The preventive basic essential newborn care specified in the action plan document mentioned drying and provision of warmth through skin to skin contact with the mother. The intervention package for the newborn in this strategy and action plan appears to focus on complication of prematurity and low birth weight, adverse intrapartum events including birth asphyxia and infection.

### Predictors of essential newborn care practices

In logistic regression analysis, the main predictors of essential new born care practices were maternal educational attainment, maternal age, and adequacy of antenatal care (ANC) attendance and maternal knowledge of newborn danger signs. Adequacy of antenatal care in particular was a consistent determinant of safe cord care, optimal thermal care and good neonatal feeding.

Maternal education of secondary school level or higher was one of the strongest socio-demographic factors that determined safe cord care, optimal thermal care practice and good neonatal feeding. This association has been reported from several countries including India and Nepal [37, 38].

The educational attainment of the mother was positively associated with the adequacy level of ANC attendance (χ2 = 59.1, *p* < 0.001) and maternal knowledge of newborn danger signs (χ2 = 16.7, p < 0.001). The effect of maternal education on essential newborn care practices was therefore mediated through ANC attendance and knowledge of newborn dangers.

We observed very strong association between use of antenatal and delivery care and the newborn care practices. This may be explained by the fact that when pregnant women make contact with health workers during ANC, they are provided with health information and made aware of proper newborn care practices. Clean cord care practice and thermal care have found to be positively associated with receiving antenatal care [37].

### Mothers’ knowledge of newborn care danger signs

Poor knowledge of newborn danger signs delays care seeking and ultimately greater risk of death and so early detection of neonatal danger signs is an important step towards improving newborn survival [39, 40]. Overall, the mothers’ knowledge of newborn care issues was not satisfactory in the sample population. In our study sample, only 45.2% of the mothers knew at least four newborn danger signs out of the seven. Knowledge on critical newborn danger signs other than high body temperature, diarrhoea, excessive crying was inadequate and similar findings have earlier been reported in the Asante Akim North District Ghana [19].

The low mothers’ knowledge of newborn care practices reported in the present study confirmed what has been reported in some other developing countries including Sri Lanka, Kenya, Uganda and Nepal had unsatisfactory level of knowledge in the recognition of newborn danger signs [34, 41–44]. Available evidence from the literature shows that the maternal knowledge is an important intermediate factor that may lead to good practices and child survival outcomes child survival outcomes [34, 41].

Though maternal knowledge of newborn danger signs was positively associated with the odds of optimal thermal care, only a small proportion of mothers were knowledgeable in newborn danger signs. This means the majority of the women lacked adequate knowledge of good newborn care practices, so their newborn practices were likely to be adversely affected. The low knowledge levels of mothers may be due to inadequate messages received on newborn care practices at the ANC. This calls for strengthening of focused health education on newborn care practices especially during prenatal care to mitigate these problems. By integrating health education on newborn care practices into routine antenatal care services, it may increase a woman’s knowledge and the ability to practice safe newborn care behaviours.

## Conclusions

The analysis from this study showed that the expected essential newborn care practices are not getting to a greater proportion of the newborns. Early bathing, non-immediate drying and application of substances to cord stump were commonly practiced. This may be depriving newborns of basic protections against infection and death.

Overall, the findings show that good neonatal feeding practices, optimal thermal care and overall adequate newborn care practices were commonly adopted by women aged 20–34 years. The results also showed that essential newborn care practices are positively associated with high maternal educational attainment, adequate utilization of antenatal care services and high maternal knowledge of newborn danger signs.

### Policy implications and recommendations

Greater improvement in essential newborn care practices could be attained through modifiable proven low-cost interventions such as effective ANC services, health and nutrition education that should span from community to health facility levels.

Of the three-composite essential newborn services indicators, optimal thermal care was the least provided to babies. Unfortunately, uptake of adequate ANC services in this study population was unrelated to optimal thermal care. This finding has important implications for the implementation of focused ANC to improve essential newborn care practices, especially that of thermal care. Therefore, ANC interventions should lay emphasis on the promotion and provision of thermal care for all newborns to prevent hypothermia (that is, immediate drying, warming, skin to skin, delayed bathing).

### Limitations of the study

There are some limitations of the findings of this study. The recall period was limited to the past 1 year in order to avoid recall bias over a longer period of time. This notwithstanding, some amount of recall bias cannot be completely ruled out. During the interviews, the women had to recall a number of events including antenatal care visits and neonatal practices. So, recall bias and misclassification might happen. In order to reduce misclassification errors, the women were required to present their antenatal records booklet for confirmation of verbal information provided.

Our design was a cross sectional study and as with all such studies, the strength of causality is weak. The cross-sectional nature of the data limits our ability to draw any causal conclusions on the relationships found in the current study. Despite these limitations, our results have shed more light on critical areas of newborn care practices that need urgent pragmatic intervention.

## Additional file

Additional file 1: The questionnaire used for the study. (PDF 172 kb)

## Abbreviations

ANC: Antenatal care
AOR: Adjusted odds ratio
MDGs: Millennium Development Goals
NMR: Neonatal mortality
PASW: Predictive analytic software
SBA: Skilled birth attendants
SDGs: Sustainable Development Goals
SES: Socio-economic statu*s*
TIBF: Timely initiation of breastfeeding
