# Supplementary material for: Prevalence and determinants of essential newborn care practices in the Lawra District of Ghana
Source: BMC Pediatr. 2018 May 24;18:173. doi: 10.1186/s12887-018-1145-4 (PMC5968597; doi:10.1186/s12887-018-1145-4)
Supplement: Supplementary file 1 — The questionnaire used for the study. (PDF 172 kb) [file 12887_2018_1145_MOESM1_ESM.pdf]

# QUESTIONNAIRE

## RELATIONSHIP BETWEEN PRENATAL CARE AND THE ADOPTION OF ESSENTIAL NEWBORN CARE PRACTICES IN THE LAWRA DISTRICT

## CONSENT

Good morning/afternoon/evening. My name is ……………………..…... a student at University for Development Studies (UDS) conducting a study on the topic " Relationship between Prenatal Care and the Adoption of Essential Newborn Care Practices in the Lawra District ". I would like to have an interview with you on the topic and would very much appreciate your participation in this study. This interview usually takes between 20 and 30 minutes to complete. All of the answers you will give will be confidential and will not be seen by anyone other than members of our team. If we should come to any question you don't want to answer, just let us know and we will go on to the next question. However, we hope you will participate fully in the survey since your views are important.

## IDENTIFICATION

District………………………………………………………………………..………..

Name of health institution…………………………………………………………..

Interview Date…………………………................ Questionnaire #..................................

House Number/Name …………………..........................................................................

Name of Interviewer………………………………………….......................................

## SECTION A: SOCIO – DEMOGRAPHIC DATA

1. Age of mother………………

2. Religion

   (A) Christianity [ ] (B) Islam [ ] (C) Traditionalist [ ]
   (D) others (specify)…………

3. Marital status

   (A) Single[ ] (B) Married[ ] (C) Divorced[ ] ( D) Widowed[ ]

4. Level of education

   (A) None [ ] (B) Primary [ ] (C) JHS [ ] (D) SHS / Vocational / Technical[ ]
   (E)Tertiary[ ]

5. Occupation of mother

   (A)Unemployed [ ] (B) Petty Trader [ ] (C) Farmer [ ] (D) Civil/Public servant (E) Others (specify)………………

6. Sex of child

   (A) Male [ ] (B) Female [ ]

7. Age of child …………….

**SECTION B: ANTENATAL CARE DURING PREGNANCY**

1. Did you ever attend antenatal clinic (ANC) during your last pregnancy?

   (A)Yes [ ] (B) No [ ]

2. How many months was your pregnancy when you first received antenatal services? .....................................................

3. How many times did you receive antenatal care during your last pregnancy? ……………………………..........

4. If the first ANC visit was initiated after the first trimester (12 weeks) what was the main reason for initiating ANC services late?..............................................................................................................................
   .....................................................................................................................................
   .....................................................................................................................................
   .....................................................................................................................................
   .....................................................................................................................................

5. Who makes the decision for you to seek prenatal care?

   (A)Husband [ ] (B) Mother in law [ ] (C) Friend [ ] (D) Self [ ]

6. Number of pregnancies? ………………………

7. Number of live births? ………………………..

8. Which of the following services did you receive from ANC. circle all that apply

(A) Weight checked at least two times

(B) Height taken on first visit

(C) Blood pressure taken at least three times

(D) Urine examination performed at least once

(E) Blood sample examination performed at least once

(F) Received health and nutrition talk at least four times on possible danger signs/complications of pregnancy

(G) Received tetanus toxoid injection at least once

(H) Received iron supplementation monthly

(I) Measurement of fundal height against the age of gestation, foetal heart beat and foetal movement count monthly

(J) Received Malaria prophylaxis at least two doses

(K) Not Applicable (Has not attended ANC)

9. How will you rate the quality/adequacy of ANC services received?

(A) Poor [ ] (B) Fair [ ] (C) Good [ ] (D) Excellent [ ] (E) Not Applicable [ ]

10. Number of tetanus toxoid (TT) injections received during the last pregnancy (Interviewer should check from the maternal health records booklet)

11. What birth preparedness and complication readiness (BPACR) practices did you follow while pregnant with the index child?

A. identified a trained birth attendant for delivery
B. identified a health facility for emergency
C. arranged for transport for delivery and/or obstetric emergency
D. saved money
E. Others (specify)…………………………………………………………..

12. Where did you deliver your child? (A) Home [ ] (B) Hospital [ ] (C) Health Centre [ ]

**SECTION C: POST NATAL CARE UPTAKE/ ESSENTIAL NEWBORN CARE PRACTICES**

1. How soon after delivery was the baby wrapped?

(A) 5 minutes [ ] (B) 5-10 minutes [ ] (C) 30 – 60 minutes [ ] (D) >60 minutes [ ]

(E)Unknown [ ]

2. When child was born, how long was it before you bathed him/her?

(A) Soon after birth [ ] (B) 1-6 hours [ ] (C) more than 6 hours but less than 24 hours [ ] (D) More than 24 hours[ ] (E) Can't tell[ ]

3. Temperature of water was used in bathing the child?

(A) Cold water [ ] (B) Hot water [ ]

4. Which of the following was used to cut the cord after delivery?
(A) New blade [ ] (B) Any available blade [ ] (C) Scissors (D) Others………………..

5. What was used to clamp the cord?
(A)Thread [ ] (B) Cord tie [ ] (C) Cord Clamp [ ] (D) String5[ ] (E) Others (specify)…………

6. What did you use to treat the cord stump?

(A) Nothing [ ] (B) Shea butter [ ] (C) Spirit [ ] (D) Shea butter with powder [ ] (E) String[ ] (F) Others………………….

7. If something was applied, what is the reason for applying that?
…………………………………………………………………………………………………
…………………………………………………………………………………………………
………………………………………………………………………………………..

8. Sometimes newborns, within the first month of life, have severe illnesses and should be taken immediately to a health facility. What types of symptoms would cause you to take your newborn to a health facility right away?

ASK: Anything else? But DO NOT READ RESPONSES. RECORD ALL THAT ARE MENTIONED

A. Convulsions ..............................
B. Fever ........................................
C. Poor suckling or feeding ...........
D. Fast/difficult breathing ..............
E. Baby feels cold ..........................
F. Baby too small/too early ...........
G. Yellow palms/soles/eyes ...........
H. Swollen abdomen ......................
I. Unconscious..............................
J. Pus or redness of the umbilical stump, eyes or skin

Other (Specify) …………………………………………………………

9. Since giving birth to the index child, have you attended postnatal care (PNC)?

(A)Yes [ ] (B) No [ ]

10. If yes, how many times have you so far attended PNC? ……………………………

11. If no, give one main reason for your action …………………………………...........

12. How many weeks after delivery of the index child did you first attend postnatal care (PNC)?

(A) Before six weeks [ ] (B) At six weeks [ ](C) After six weeks[ ] (D) Not applicable [ ]

**SECTION D: INFANT AND YOUNGCHILD FEEDING PRACTICES.**

1. What time did you initiate breastfeeding?

   (a) Within the first 30 minutes [ ] (b) After 30 minutes [ ] (c) The next day [ ]

   (d) Others specify……………………………………………………………….

2. Who assisted you in the process?

   (A) A midwife/nurse [ ] (B) A relative [ ] (C) myself [ ]

   (E) Others specify …………………………………..

3. Are you still breastfeeding your child? (A) Yes [ ] (b) No [ ] if yes move to the next question. If yes skip to question 6

3b. If (child's name) is not currently breastfeeding, how many months did you breastfeed him/her?

(A) Less than six months [ ] (B) 6-12 months [ ] (C) 13-23 months [ ]

4. Before putting (Name of child) to the breast for the first time after delivery, what was child given to drink? (Multiple responses possible)
   1. Nothing
   2. Milk (other than breast milk)
   3. Plain water
   4. Sugar or glucose water
   5. Gripe water
   6. Sugar-salt-water solution
   7. Fruit juice
   8. Infant formula
   9. Tea / coffee
   10. Honey
   11. Other (specify) _____________

5. When you delivered (Name of child) what did you do with the first yellowish breast milk?

(A) Gave it to the baby [ ] (B) Discarded it [ ] (C) Other (Specify) _____________

6. Yesterday, was [child's name] breastfed?

(A) Yes [ ] (B) No [ ] (C) Not Applicable [ ]

7. Yesterday did [child's name] have anything to drink from a bottle with a nipple during the day or night? (A) Yes [ ] (B) No [ ]

8. Kindly mention all liquids (child’s name) drank yesterday during the day or at night **(Multiple responses possible)**

1. Nothing
2. Breast milk
3. Plain water
4. Commercially produced infant formula (e.g. Lactogen or SMA)
5. Any other milk such as evaporated/sweetened condensed milk, powdered, or fresh animal milk
6. Sugar water, coconut, pito, other fruit juice or canned drink
7. Tea or coffee
8. Liquid or semi-liquid traditional medicine
9. Other liquid (specify) ___________________________________

9. At what age did you first give solid or semisolid food to [child’s name]?

1. Before 6 months
2. At Six months
3. Seven to 9 months
4. After nine months
5. Yet to start

10. What was the first solid or semi-solid food given to (Child’s Name)?

1. Mashed kenkey [ ]
2. Soft banku, [ ]
3. Koko, [ ]
4. Soft tuo zaafi, [ ]
5. Mashed yam [ ]
6. Weanimix [ ]
7. Other (Specify)……………………………………………………

11. Yesterday did [child’s name] eat any solid or semi-solid foods?

1. Yes [ ]
2. No [ ]
3. Does not apply (child does not eat solid foods) [ ]

4. Does not know [ ]

12. How many times did (Name of child) eat solid or semi-solid food or soft foods other than liquids yesterday during the day or at night? …………………………….

13a) Please, mention all the foods and drinks that were eaten by (Name of child) over the past 24 hours whether at home or outside the home. (Hint: start with meal eaten at supper yesterday).

| Eating moment | Name of dish | Ingredients |
|---|---|---|
| Breakfast | | |
| Snack before lunch | | |
| Lunch | | |
| Snack before dinner | | |
| Dinner | | |

| | | |
|---|---|---|
| Snack after dinner | | |
| Drinks | | |

13b.From the meals mentioned by the mother, indicate whether (Name of child), ate from the following food groups during the past 24 hours whether at home or outside the home.

| **Food group** | **Examples** | **YES** | **NO** |
|---|---|---|---|
| CEREALS | Bread, noodles, biscuits, any other food made from millet, sorghum, maize, rice, wheat. | | |
| WHITE TUBERS AND ROOTS | White potatoes, white yam, cassava, or food made from roots. | | |
| DARK GREEN LEAFY VEGETABLES | Dark green leafy vegetables, including wild ones + other locally available vitamin-A rich leaves such as cassava leaves, ayoyo, alefu, bra, fresh baobab leaves etc. | | |
| VIITAMIN A RICH VEGETABLES AND TUBERS | carrots, sweet potatoes that are orange inside + other locally available vitamin –A rich vegetables (e.g. Sweet pepper) | | |
| FRESH VITAMIN A RICH FRUITS | Ripe mangoes, papayas, dawadawa pulp (yellow part) + other locally available vitamin-A rich fruits | | |
| DRIED FRUITS AND VEGETABLES | Any form of dried vegetables (okro, baobab leaves (kuuka), wild types | | |
| ORGAN MEAT (IRON-RICH) | Liver, kidney or other organ meats or blood-based foods | | |
| FLESH MEATS | Beef, pork, lamb, goat, rabbit, wild game, chicken, duck, or other birds | | |
| EGGS | fowl, duck, guinea fowl or any other egg | | |
| FISH | keta schoolboys (anchovies), tilapia, mudfish etc | | |
| LEGUMES, NUTS, AND SEEDS | Beans, pigeon peas, soya beans, groundnuts, bambara nuts, bungu, neri, | | |
| MILK AND MILK PRODUCTS | Milk, cheese (wagashi), yogurt or other milk products | | |
| OILS AND FATS | groundnut oil, palm oil, sheabutter, margarine | | |
| SPICES CONDIMENTS, BEVERAGES | Spices (black pepper, salt), condiments(e.g.dawadawa, kanton, maggi),coffee, tea, alcoholic beverages e.g. pito | | |

## SECTION E: CHILD MORBIDITY AND UTILIZATION OF HEALTH SERVICES

1. Has (Name of child) had an illness with a cough that comes from the chest at any time in the last two weeks?

(1).Yes [ ]  (2) No [ ]  (3) Don't know [ ]

2. Did (Name of child) get diarrhea in the past two weeks? ( Diarrhoea is having loose watery stools more than 3 times).  (1) Yes [ ]  (2) No [ ]  (3) Don't know [ ]

3. When (name of child) had diarrhoea, what treatment was given?

1. Nothing
2. ORS
3. ORS Plus Zinc
4. Sugar-salt solution
5. Infusion at the hospital
6. Other (specify)……………………………………………
7. Not Applicable (Child had no diarrhoea)

4. The last time [child's name] was sick, did you offer less, more or the same amount of breast milk as when [child's name] is healthy? (If response is "less", ask additional questions to determine why.)

1. Less, because the child did not want it
2. Less, because mother's decision
3. More
4. The same
5. Child never breastfed or child stopped breastfeeding before last illness
6. Child has never been sick (skip to question 6)
7. Does not know

5. The last time [child's name] was sick, did you offer less, more or the same amount of solid/semi-solid foods as when [child's name] is healthy? IF THEY RESPOND "LESS" THEN PROBE "WHY?")

1. Less than usual, because the child did not want it
2. Less than usual, because mother's decision
3. More than usual
4. The same as usual
5. Stopped completely
6. Child has never been sick
7. Does not know
8. Not applicable

6. During the past 6 months, did [child's name] ever take a vitamin A capsule? (Verify from child's records booklet)

(A) Yes [ ] (B) No [ ](C) Does not know[ ] (D) Not applicable[ ]

7. Record from the Child Health Record Card the number of times in the last 4 months (Name of child) was weighed ..................................

8. Do you have a card where (name of child) vaccinations are written down?

(A) Yes, seen by interviewer

(B) Not available/lost/misplaced

(C) Never had a card

If yes request for it and check the record booklet and record the dates of vaccination given

| Vaccinations | Date Received | comments |
|---|---|---|
| BCG | | |
| Polio 0 | | |
| Polio 1 | | |
| Polio 2 | | |
| Polio 3 | | |
| Penta 1 | | |
| Penta 2 | | |
| Penta 3 | | |
| Measles | | |
| Yellow fever | | |
| Rotavirus | | |
